# Tuberculin Test in New York State

**Published:** 1898-08

**Authors:** 


New York State is now realizing the force of the action of Massachusetts and Pennsylvania requiring tuberculin-tested cattle free from tuberculosis to replenish their dairies, and the State Board of Health are more than apprehensive over the prospects of rejected cattle being sold in the Empire State. Well she may be, and if her people do not adopt some broader methods beyond the picayune measures now prevailing, she will find that the time will soon come when she will be sorely taxed in a financial direction to do this work, so pressing over our own land and the civilized world. A more aroused public, growing better informed daily, will not tolerate much longer a continual record of fourteen deaths out of every one hundred in the civilized world, and 10 to 15 per cent. of the dairy cows suffering with the same disease, yet supplying as a raw product one of the principal foods of mankind.

